# Effect of chronic kidney disease on total knee arthroplasty outcomes: a meta-analysis of matched control studies

**DOI:** 10.1186/s42836-021-00078-4

**Published:** 2021-07-02

**Authors:** Chongjie Cheng, Yan Yan, Qidong Zhang, Wanshou Guo

**Affiliations:** 1 Graduate School of Peking Union Medical College, Beijing, China; 2 China-Japan Friendship Institute of Clinical Medicine, Beijing, China; 3 Department of Orthopaedic Surgery, China-Japan Friendship Hospital, Beijing, China; 4 Peking University China-Japan, Friendship Institute of Clinical Medicine, Beijing, China

**Keywords:** Total knee arthroplasty, Chronic kidney disease, Perioperative complication, Length of stay

## Abstract

**Purpose:**

The purpose of this meta-analysis was to review the current evidence in the literature to find out whether the coexisting chronic kidney disease affected infection, revision, transfusion, readmission, mortality, and the length of hospital stay after total knee arthroplasty.

**Methods:**

Medline, PubMed, Embase, and the Cochrane Library were searched from their dates of inception to June 30, 2020. The primary outcomes were postoperative infection, revision, and mortality. The secondary outcomes were transfusion, the length of hospital stay, and readmission. A *P* value of < 0.05 was deemed to be statistically significant.

**Results:**

A total of 881 articles were identified, and 7 articles that met the inclusion criteria were identified to be eligible. The most important finding of our study was that the chronic kidney disease was associated with increased postoperative transfusion (*P* < 0.05) and mortality (*P* < 0.05). Meanwhile, the patients with chronic kidney disease were associated with a higher readmission rate, compared to the patients without chronic kidney disease (*P* < 0.05). However, chronic kidney disease was not associated with high risks for infection (*P* > 0.05), revision surgeries (*P* > 0.05), and a prolonged hospital stay (*P* > 0.05).

**Conclusions:**

After total knee arthroplasty, the patients with coexisting chronic kidney disease carry higher risks of transfusion, mortality, and readmission. However, the chronic kidney disease may not be associated with the risk of infection or revision, nor the duration of hospitalization.

## Introduction

Total knee arthroplasty (TKA) is a common surgical procedure for the end-stage knee osteoarthritis [1, 2], while the prevalence of chronic kidney disease (CKD) is increasing worldwide [3]. Recent studies suggest that the TKA patients with CKD carry higher risks of postoperative complications [4–6]. However, a single study compromises the reliability of the conclusions due to its relatively small amount of data and statistical power.

Kidney function is defined using the estimated glomerular filtration rate (GFR). The severity is classified into five stages based on the level of estimated GFR (stage I, > 90 mL/min; stage II, 60–89 mL/min; stage III, 30–59 mL/min; stage IV, 15–29 mL/min; stage V renal failure, < 15 mL/min) [7]. Patients with end-stage CKD must receive dialysis or kidney transplantation, which augment the potential risks of complications after TKA. In addition, the CKD is associated with the comorbidities such as anemia, diabetes mellitus, hypertension, weakened immune system. Each of them is an independent risk factor for postoperative complications [8]. DiMagno *et al*. [3] found that patients with stage III, IV, or V CKD are at greater risks of complications after TKA. Gwam *et al*. [4] found the dialysis-dependent patients are associated with a prolonged hospital stay, but do not carry the risk of 30-day complications. Antonia *et al*. [5] suggested stage III and IV CKD were associated with the major postoperative complications. Currently, no meta-analysis elucidated the outcomes of TKA in CKD patients.

The purpose of this meta-analysis was to review the current evidence in the literature to find out whether the CKD affects the infection, revision, transfusion, readmission, mortality, and the length of stay (LOS) after TKA. The primary outcomes were infection, revision, and mortality. The secondary outcomes were transfusion rate, the length of hospital stay, and readmission. This is the first meta-analysis to assess the outcomes of TKA in CKD patients.

## Materials and Methods

### Search strategy

The systematic literature review was structured to adhere to the Preferred Reporting Items for Systematic Reviews and Meta-Analyses statement, which included the requirements deemed essential for the transparent reporting of results [9]. Ethical approval was not needed because all the data presented in this study were extracted from the published articles and did not cover any personal data. We searched the online databases including Medline, PubMed, Embase, and the Cochrane Library from their dates of inception to June 30, 2020. We searched the following terms: (Renal Insufficiency OR Kidney Insufficiency OR Kidney Failure OR Renal Failure OR Kidney Failure, Chronic OR End-Stage Kidney Disease OR End-Stage Renal Disease OR Dialysis OR Hemodialysis) AND (Arthroplasty, Replacement, Knee OR Arthroplasty, Knee Replacement OR Knee Replacement Arthroplasty OR Knee Arthroplasty, Total OR Total Knee Arthroplasty OR Total Knee Replacement OR Knee Arthroplasty OR Arthroplasty, Knee OR Arthroplasties, Knee Replacement OR Replacement Arthroplasty, Knee). Publication language was limited to English. The references of the selected articles were also searched and reviewed.

### Eligibility criteria

The articles included in the meta-analysis met all of the following inclusion criteria (in the PICOS order): (1) population: patients undergoing primary TKA or minimally invasive TKA; (2) intervention: CKD group; (3) comparison intervention: non-CKD group; (4) outcome measures: at least one of the following outcomes was reported: infection, revision, transfusion, the length of stay (LOS), re-admission, and mortality; (5) study design: prospective study, retrospective study or registry study. The articles that did not assessthe outcomes mentioned above were excluded. The articles evaluating the revision surgery or unicompartmental knee arthroplasty, involving no comparison between CKD and non-CKD or combining outcomes of TKA and total hip arthroplasty were excluded. The conference abstracts, case reports, biochemical trials, letters, and reviews were also excluded.

### Data extraction

Two reviewers (CJ and YY) independently checked the full text of the selected articles. Raw information including characteristics of the articles (author, publication year, study design, and follow-up period) and patients’ demographic details (the number of patients, average age, gender ratio, and stage of CKD) were extracted. The primary outcomes were the incidence of postoperative complications, including dislocation, infection, deep vein thrombosis (DVT), pulmonary embolism (PE), revision surgeries, and mortality. The secondary outcomes included re-admission, transfusion, and duration of hospitalization. If data were incomplete or could not be extracted directly, we contacted the corresponding authors to ensure the integrity of the data.

### Statistical analysis

We used the Review Manager software (v 5.3; Cochrane Collaboration) to perform the meta-analysis. The extracted data were independently entered into the Review Manager by one author and independently checked by another author. The results of the selected studies were pooled for meta-analysis when two or more results were available. We applied the Mantel-Haenszel method to compute the pooled odds ratio (OR). An OR with a 95% confidence interval (CI) or a mean difference with a 95% CI was categorized as dichotomous outcomes or continuous outcomes, respectively. A *P* value of < 0.05 was deemed to be statistically significant. We used the *Q* and chi² tests to estimate the *P* value and *I*² of the heterogeneity measures. All outcomes were pooled on the random-effect model.

### Quality evaluation

Because no randomized trials were included, the Newcastle-Ottawa scale (0 = very poor to 9 = rigorous) was applied to evaluate the quality of non-randomized studies. The Newcastle-Ottawa scale-based methodological quality assessment was conducted in three domains: study selection, intergroup comparability, and exposure (Table 1). A higher score indicated a better quality of an article. Two reviewers (CJ and YY) independently assessed the included articles, and disagreements were resolved by discussion with a third reviewer (WS). The sensitivity analysis was also conducted to evaluate whether any single study had the weight to skew on the whole estimate and data. Publication bias analyses were not performed due to the relatively scant literature.

**Table 1:** Newcastle-Ottawa scale

Study	Is the case definition adequate?	Representativeness of the cases	Selection of Controls	Definition of Controls	Comparability of cases and controls on the basis of the design or analysis	Ascertainment of exposure	Same method of ascertainment for cases and controls	Non-Response rate	Scores
McCleery 2010	1	1	1	1	2	1	1	1	9
Miric 2013	1	1	1	1	1	1	1	1	8
Ponnusamy 2015	1	0	1	1	0	1	1	1	6
Lizaur-Utrilla 2016	1	0	1	1	0	1	1	1	6
Kuo 2016	1	1	1	1	1	1	1	1	8
Kuo 2017	1	1	1	1	1	1	1	1	8
Bedard 2018	1	0	1	1	0	1	1	1	6

## Results

### Search results and study characteristics

**Fig. 1:**
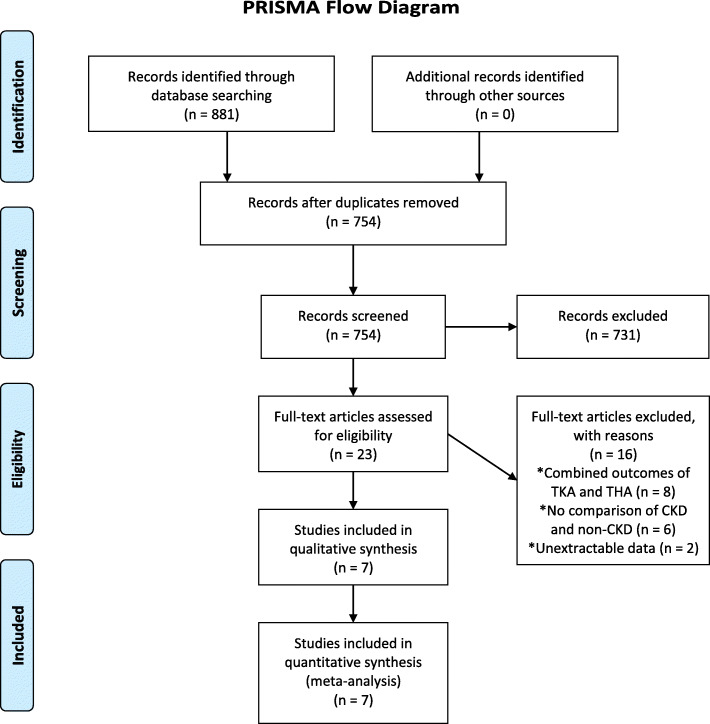
PRISMA flowchart. A total of 881 studies were evaluated. Titles and abstracts were assessed, and 23 full-text articles were eligible for evaluation. Sixteen articles were excluded, and 7 articles remained for the final analysis. TKA: total knee arthroplasty; THA: total hip arthroplasty; CKD: chronic kidney disease

A total of 881 articles were initially identified from the online registerry databases, and 858 articles were excluded after primary review of the titles and abstracts. After further full-text evaluation, 16 articles that did not meet the inclusion criteria were excluded. Finally, 7 articles were included in the meta-analysis (Fig. 1) [6, 10–15]. The baseline characteristics of the studies and patient demographic details are shown in Table 2. Among them, 6 articles [6, 10–12, 14, 15] were defined as having CKD using the international classification of diseases (ICD) or diagnostic codes on their electronic medical records or registry databases. One article [13] was defined as having CKD by calculating the estimated GFR (< 60 mL/min/1.73 m^2^ ; stage II-V). All 7 studies were matched control studies. The Newcastle-Ottawa scales of the included studies ranged from 6 to 9, indicating that the quality of the included studies was at the upper-middle level, and the outcomes of the included studies could be considered to be reliable (Table 1). The 7 studies included a total of 4,329,455 patients. Among them, 14,515 patients (TKA group; 0.33 %) suffered from chronic kidney disease prior to TKA, and 4,314,940 patients (no-TKA group; 99.66 %) had not. The mean age of the two groups were similar. Of the 7 studies, the percentage of female patients ranged from 27 to 77 %, and the mean follow-up periods lasted 2 to 3.4 years.

**Table 2:** Characteristics of the included studies

Study	Study Design	No. of Patients	CKD	Non-CKD	Mean Age (y)		Female Gender (%)	Follow-Up mean (range) (y)	Stage of CKD	Method to identify CKD	Outcome Measures
					CKD non-CKD						
McCleery 2010	MR	59,288	3916	55,372	NC	NC	58.9	NC	Stage 1–5, including dialysis and transplant	ICD	Infection, revision;
Miric 2013	MR	36,882	2686	34,196	73	67	62.4	2.1 (0–5)	Stage 3 (69 %), stage 4 (3 %), stage 5 (5.5 %), unspecified stage (22 %), including transplant	ICD	Infection, revision, PE, DVT, mortality, re-admission;
Ponnusamy 2015	MR	4,182,887	1683	4,181,204	66.7	66.8	63.8	NC	Stage 5, with dialysis	ICD	Infection, mortality, transfusion;
Lizaur-Utrilla 2016	MR	45	15	30	69.3	70.1	53.3	3.4 (2–6)	Stage 5, including dialysis and transplant	Using diagnostic and surgical codes on the departmental arthroplasty database	Infection, transfusion;
Kuo 2016	MR	615	205	410	72.1	71.0	27.3	2.7 (2-4.8)	Stage 2–5 (eGFR < 60 mL/min/1.73 m^2^)	Calculating eGFR	LOS, transfusion;
Kuo 2017	MR	13,844	1459	12,385	71.6	70.3	76.8	2	Stage 1–5	ICD	Infection, PE, DVT, mortality, re-admission;
Bedard 2018	MR	35,894	4551	31,343	NC	NC	63.7	2	Stage 1–5	ICD	Revision;

*MR* matched retrospective; *CKD* chronic kidney disease; *NC* not clear; *LOS* length of stay; *PE* pulmonary embolism; *DVT* deep vein thrombosis; *ICD* International Classification of Diseases; *eGFR* estimated glomerular filtration rate

### Meta-analyses

**Fig. 2:**
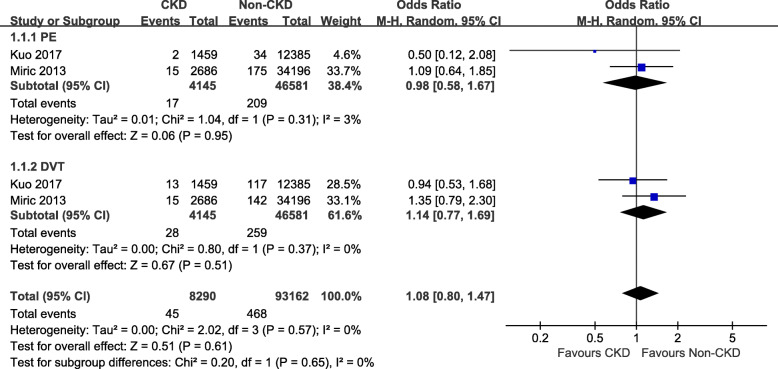
Forest plots of the VTE (both PE and DVT) between CKD group and non-CKD group after TKA

**Fig. 3:**
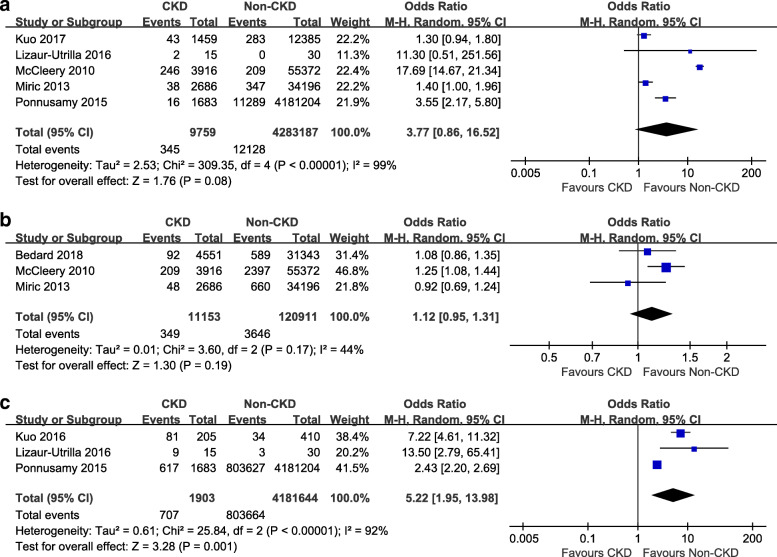
(**a**) Forest plots of the infection between CKD group and non-CKD group after TKA; (**b**) Forest plots of the revision between CKD group and non-CKD group after TKA; (**c**) Forest plots of the transfusion between CKD group and non-CKD group after TKA

**Fig. 4:**
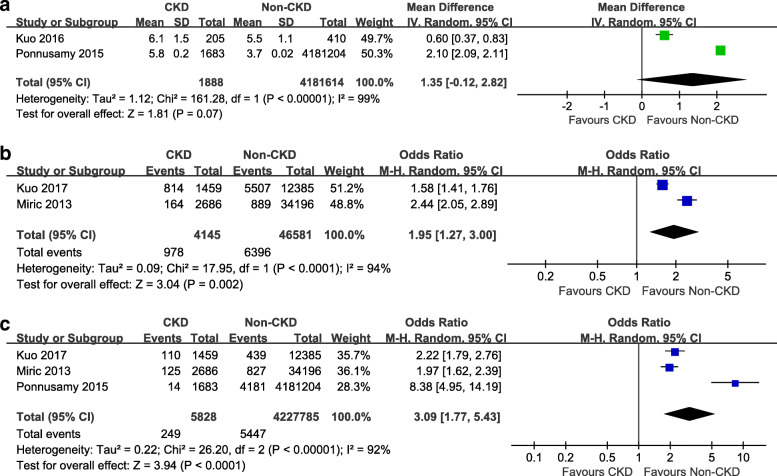
(**a) **Forest plots of the length of stay between CKD group and non-CKD group after TKA; (**b**) Forest plots of the re-admission between CKD group and non-CKD group after TKA; (**c**) Forest plots of the mortality between CKD group and non-CKD group after TKA

Venous thrombus embolism (PE and DVT) were reported in 2 studies [6, 14], the incidences of VTE were 1.09 % in CKD group and 1 % in non-CKD group (OR 1.08, 95 % CI 0.80–1.47, *P* = 0.61) (Fig. 2). Postoperative infection was reported in 5 studies [6, 10–12, 14], but there was no significant difference between the groups (OR 3.77, 95 % CI 0.86–16.52, *P* = 0.08) (Fig. 3a). Revision surgeries were reported in 3 studies [6, 10, 15], but there was no significant difference between the groups (OR 1.12, 95 % CI 0.95–1.31, *P* = 0.19) (Fig. 3b). Blood transfusion was reported in 3 studies [11–13], and the incidences were 37 % (707 of 1903 patients) in CKD group and 19 % (803,664 of 4,181,644 patients) in non-CKD group (OR 5.22, 95 % CI 1.95–13.98, *P* = 0.001) (Fig. 3c). The LOS was assessed in 2 studies [11, 13], including 4,183,502 patients. There was no significant difference between the groups (mean difference = 1.35, 95 % CI –0.12–2.82, *P* = 0.07) (Fig. 4a). Re-admission was assessed in 2 studies [6, 14]. The pooled results showed that the CKD group had a higher re-admission rate than the non-CKD group (OR 1.95, 95 % CI 1.27–3.00, *P* < 0.0001) (Fig. 4b). Postoperative mortality was assessed in 3 studies [1, 11, 14]. The pooled results indicated that the CKD group was associated with higher postoperative mortality, compared to the non-CKD group (OR 3.09, 95 % CI 1.77–5.43, *P* < 0.0001) (Fig. 4c). Sensitivity analysis was conducted among the outcomes with a high heterogeneity, and showed that removing any single study did not change the statistical results.

## Discussion

Recent studies showed that the prevalence rates of CKD in TKA patients are between 6 % and 27 % [16, 17]. In this study, we found the CKD patients carried higher risks of postoperative mortality and re-admission, compared to the non-CKD patients. The CKD patients had a higher probability of postoperative transfusion than their non-CKD counterparts. However, the CKD did not affect the risks of postoperative infection, revision or LOS.

Ponnusamy *et al*. [11] found the CKD affected bone volume, mineralization, linear growth, and strength [18]. Tan *et al*. [8] demonstrated the patients with end-stage CKD carried a higher risk of VTE, because the increased inflammatory state produces the hypercoagulability [19]. The chronic inflammation produces excessive fluid in the soft tissue surrounding the knee and increases the risks of periprosthetic infection and revision surgery [20]. The CKD is often associated with a poor nutritional status, electrolyte disorders, decreased immunity, and anemia [1, 8, 21, 22]. Those factors also increase the risk of postoperative infection. McCleery *et al*. [10] reported that the CKD increased the risk of early periprosthetic infection by 50 %.

However, there are different conclusions. Chen *et al*. [1] retrospectively evaluated 15 CKD patients (18 knees) who underwent TKA with antibiotic-loaded cement, and no infection was reported after a mean follow-up period of 25 months. Ling *et al*. [23] evaluated 13 CKD patients (18 knees) who underwent TKA, and no infection was found after a mean follow-up period of 5 years. Warth *et al*. [16] found that there were no differences between the CKD patients and non-CKD patients regarding the infectious surgical site complications, such as superficial or deep wound infection, organ space infection or wound dehiscence. Several studies revealed the combined use of antibiotic-loaded bone cement and systemic antibiotic therapy can decrease the risk of postoperative infection, but similar infection rates were reported in both CKD and non-CKD patients [1]. Wang *et al*. [24] did not find an increased infection rate even in the patients who experienced a long period of dialysis vintage.

McCleery *et al*. [10] found the patients on dialysis had an increased revision rate within one year after TKA, but the result is similar to the revision rate of non-CKD patients. However, Miric *et al*. [6] found there was no difference between the early and later revision rates. The most common cause of revision TKA is the periprosthetic joint infection, accounted for 62 % of all revision TKAs [25]. We found the similar revision rates in the CKD and the non-CKD patients. However, confirming the actual causes of revision is often difficult, which affects the assessment of the actual revision rates.

Patients with CKD and on long-term dialysis are often associated with renal anemia, metabolic imbalance, elevated risk of bleeding, and poor vascular circulation [26]. Graves *et al*. [27] showed that the degree of anaemia was more pronounced in patients with more advanced CKD. Moreover, the intra- and postoperative blood loss ranges from 800 mL to 1000 mL [28]. Kaiser *et al*. [29] found the CKD patients had a higher rate of transfusion (24 %) compared to non-CKD patients (8 %). Our study also supports this conclusion.

Death is a rare and devastating complication of TKA, but the early reported mortality rates range from 17 to 58 % in CKD patients [6, 30, 31]. Ponnusamy *et al*. [11] reported athe mortality rate of 1 % in dialysis-dependent patients. Several articles reported that the CKD is the independent risk factor of mortality in the early 90 days, and is the independent risk factor of morbidity in the early 30 days [13, 32, 33]. Warth *et al*. [16] demonstrated the incremental mortality was associated with an increased risk of postoperative pulmonary and cardiovascular complications. Our meta-analysis supports these findings.

Our sensitivity analysis was conducted among the outcomes with a high heterogeneity. Removing any single study  did not change the statistical results. Therefore, we believe our findings in this meta-analysis are reliable.

Our meta-analysis has limitations. First, the retrospective studies included may lead to potential biases. Second, analyzing all CKD patients together without considering the severity may cause sampling bias, because there is a dose-dependent relationship between the severity of CKD and outcomes of TKA [6]. Third, owing to the relatively short duration of follow-up, infection and revision rates were potentially underestimated. Fourth, the presence of publication bias is likely to decrease our confidence in the meta-analytic findings [12]. Fifth, each comorbidity (diabetes, heart failure, peripheral vascular disease, hypertension, *etc*.) is analysed as an independent risk factor, but overlooking the fact that the patients with multimorbidities may lead to selection bias [3]. Sixth, in the future, more high quality and matched control studies should be conducted to improve the statistical efficiency and precision.

## Conclusions

Compared to the non-CKD patients, CKD patients carry higher risks of mortality and re-admission after TKA, as well as a higher incidence of transfusion. However, the CKD may not increase the risk of infection or revision, nor the duration of hospitalization.

## Data Availability

All data generated or analyzed during this study are included in this published article and its additional information files.

## Abbreviations

TKA: Total knee arthroplasty
CKD: Chronic kidney disease
LOS: Length of stay
VTE: Venous thrombus embolism
PE: Pulmonary embolism
DVT: Deep vein thrombosis
ICD: International classification of diseases
GFR: Glomerular filtration rate

## References

[1] Wang J, Chen J, Kuo F (2014). Total knee arthroplasty in patients with dialysis: Early complications and mortality. Biomedical Journal.

[2] Hunter DJ, Bierma-Zeinstra S (2019). Osteoarthritis The Lancet.

[3] DiMagno AN (2020). Chronic kidney disease impact on total joint arthroplasty outcomes: A National Inpatient Sample-based study. Journal of Orthopaedic Surgery.

[4] Gwam CU (2020). Dialysis Is Not Associated with Increased Risk of Perioperative Complications in TKA Patients after Adjusting for Pertinent Confounders. The Journal of Knee Surgery.

[5] Antoniak DT (2020). Impact of Chronic Kidney Disease in Older Adults Undergoing Hip or Knee Arthroplasty: A Large Database Study. The Journal of Arthroplasty.

[6] Miric A, Inacio MC, Namba RS (2013). Can total knee arthroplasty be safely performed in patients with chronic renal disease?. Acta Orthop.

[7] Jämsä P (2018). Moderate to Severe Renal Insufficiency Is Associated With High Mortality After Hip and Knee Replacement. Clin Orthop Relat Res.

[8] Tan TL (2016). Chronic Kidney Disease Linearly Predicts Outcomes After Elective Total Joint Arthroplasty. The Journal of Arthroplasty.

[9] Moher D (2009). Preferred reporting items for systematic reviews and meta-analyses: the PRISMA statement. BMJ.

[10] McCleery MA, Leach WJ, Norwood T. Rates of infection and revision in patients with renal disease undergoing total knee replacement in Scotland. The Journal of Bone and Joint Surgery. British volume, 2010. 92-B(11): p. 1535–1539. 10.1302/0301-620X.92B11.23870 21037348

[11] Ponnusamy KE (2015). Inpatient Mortality and Morbidity for Dialysis-Dependent Patients Undergoing Primary Total Hip or Knee Arthroplasty. The Journal of Bone Joint Surgery-American Volume.

[12] Lizaur-Utrilla A (2016). Elective Total Knee Arthroplasty in Patients With End-Stage Renal Disease: Is It a Safe Procedure?. The Journal of Arthroplasty.

[13] Kuo F (2017). Chronic Kidney Disease Is an Independent Risk Factor for Transfusion, Cardiovascular Complication, and Thirty-Day Readmission in Minimally Invasive Total Knee Arthroplasty. The Journal of Arthroplasty.

[14] Kuo LT (2017). Chronic kidney disease is associated with a risk of higher mortality following total knee arthroplasty in diabetic patients: a nationwide population-based study. Oncotarget.

[15] Bedard NA (2018). Preoperative Opioid Use and Its Association With Early Revision of Total Knee Arthroplasty. The Journal of Arthroplasty.

[16] Warth LC (2015). Total Joint Arthroplasty in Patients with Chronic Renal Disease: Is It Worth the Risk?. The Journal of Arthroplasty.

[17] Ackland GL (2011). Chronic Kidney Disease and Postoperative Morbidity After Elective Orthopedic Surgery. Anesthesia Analgesia.

[18] Ottesen TD (2018). Dialysis Patients Undergoing Total Knee Arthroplasty Have Significantly Increased Odds of Perioperative Adverse Events Independent of Demographic and Comorbidity Factors. The Journal of Arthroplasty.

[19] Li Q, et al., Chronic Kidney Dysfunction Can Increase the Risk of Deep Vein Thrombosis after Total Hip and Knee Arthroplasty. BioMed Research International, 2017. 2017: p. 1–6. 10.1155/2017/8260487 PMC5458429 28612028

[20] Muratli HH (2005). Simultaneous rupture of the quadriceps tendon and contralateralpatellar tendon in a patient with chronic renal failure. Journal of Orthopaedic Science.

[21] Li M (2019). Red blood cell distribution width and platelet counts are independent prognostic factors and improve the predictive ability of IPI score in diffuse large B-cell lymphoma patients. BMC Cancer.

[22] Dong B (2014). A retrospective study of cytomegalovirus pneumonia in renal transplant patients. Exp Ther Med.

[23] Ling ZM (2016). Low Infection Rates in Total Knee Arthroplasty in End Stage Renal Failure Patients. The Journal of Arthroplasty.

[24] Wang Y (2019). Is dialysis vintage a perioperative risk for end-stage renal disease patients receiving total knee and hip arthroplasty. Journal of Orthopaedic Surgery.

[25] Urish KL (2020). Comparison of readmission and early revision rates as a quality metric in total knee arthroplasty using the Nationwide Readmission Database. Annals of Translational Medicine.

[26] Cavanaugh PK (2016). Complications and Mortality in Chronic Renal Failure Patients Undergoing Total Joint Arthroplasty: A Comparison Between Dialysis and Renal Transplant Patients. The Journal of Arthroplasty.

[27] Graves A (2014). Predictors of perioperative blood transfusions in patients with chronic kidney disease undergoing elective knee and hip arthroplasty. Nephrology.

[28] Seo J (2013). The comparative efficacies of intra-articular and IV tranexamic acid for reducing blood loss during total knee arthroplasty. Knee Surg Sports Traumatol Arthrosc.

[29] Kaiser C (2019). The influence of chronic kidney disease on the duration of hospitalisation and transfusion rate after elective hip and knee arthroplasty. Int Urol Nephrol.

[30] Sunday JM, Guille JT, Torg JS (2002). Complications of Joint Arthroplasty in Patients with End-Stage Renal Disease on Hemodialysis. Clin Orthop Relat Res.

[31] Bozic KJ (2012). Patient-related Risk Factors for Postoperative Mortality and Periprosthetic Joint Infection in Medicare Patients Undergoing TKA. Clinical Orthopaedics Related Research®.

[32] Bozic KJ (2012). Patient-Related Risk Factors for Periprosthetic Joint Infection and Postoperative Mortality Following Total Hip Arthroplasty in Medicare Patients. The Journal of Bone Joint Surgery-American Volume.

[33] Belmont PJ (2014). Morbidity and Mortality in the Thirty-Day Period Following Total Hip Arthroplasty: Risk Factors and Incidence. The Journal of Arthroplasty.

